# Comparison of Implant Stability between Regenerated and Non-Regenerated Bone. A Prospective Cohort Study

**DOI:** 10.3390/jcm10153220

**Published:** 2021-07-21

**Authors:** Marta Vallecillo-Rivas, Candela Reyes-Botella, Cristina Vallecillo, María Jesús Lisbona-González, Manuel Vallecillo-Capilla, María Victoria Olmedo-Gaya

**Affiliations:** 1 Department of Oral Surgery and Implantology, Faculty of Dentistry, University of Granada, Colegio Máximo de Cartuja s/n, 18071 Granada, Spain; mvallecillo@correo.ugr.es (M.V.-R.); creyes@ugr.es (C.R.-B.); cvallecillorivas@hotmail.com (C.V.); mjlisbona@icloud.com (M.J.L.-G.); mvolmedo@ugr.es (M.V.O.-G.); 2 Medicina Clínica y Salud Pública PhD Programme, Faculty of Dentistry, University of Granada, 18071 Granada, Spain; 3 Department of Oral and Maxillofacial Surgery, Faculty of Dentistry, University of Granada, 18071 Granada, Spain

**Keywords:** bone quality, bone regeneration, dental implants, implant stability, osseointegration, resonance frequency analysis (RFA), xenograft

## Abstract

Implant stability is one of the main indicators of successful osseointegration. Although it has been measured in numerous studies, there has been little research on implant stability in regenerated bone. The study compares primary and secondary stability between implants placed in regenerated versus native bone and evaluates the influence of bone quality on the results. Sixty implants were placed in 31 patients: 30 implants inserted in native bone (non-regenerated) after a healing period of at least 6 months post-exodontia and 30 inserted in regenerated bone at 6 months after grafting with xenograft. Resonance frequency analysis (RFA) was used to obtain implant stability quotient (ISQ) values at baseline (implant placement), 8 weeks, and 12 weeks. Statistically significant differences were found between implants placed in regenerated bone and those placed in native bone at all measurement time points (*p* < 0.05). ISQ values were significantly influenced by bone quality at baseline (*p* < 0.05) but not at 8 or 12 weeks. Greater stability was obtained in implants placed in native bone; however, those placed in regenerated bone showed adequate primary and secondary stability for prosthetic loading. Bone quality influences the primary but not secondary stability of the implants in both native and regenerated bone.

## 1. Introduction

Osseointegration was first defined by Brånemark in 1976 [1] as the direct and long-lasting anchorage between patient bone and the implant surface. Albrektsson recently described osseointegration as the formation of new bone from a defense reaction against a foreign body [2,3]. Osseointegration is clinically and histologically determined by the stability of the implant, i.e., the absence of movement [4]. An important differentiation is made between primary and secondary stability. Primary stability, or the mechanical connection between bone and implant [5], is the stability immediately after implant placement and depends on the bone quality, macroscopic implant design, and surgical procedure [6,7]. Subsequent maturation of the bone in direct contact with the implant surface gives rise to secondary or biological stability [8,9], which depends on the microscopic implant design and its physical properties [3].

Over the past few years, resonance frequency analysis (RFA) has been used to determine implant stability [10,11]. This non-invasive diagnostic technique yields a quantitative estimation of implant stability by applying magnetic impulses on a metallic transducer (peg) previously screwed to the implant and transforming them into an electromagnetic response, measured in KHz [12]. This response is expressed as the implant stability quotient (ISQ) value, which runs from 1 to 100 units [13,14,15], and where higher values indicate greater stability [16].

The main objectives of implantology research have long been to determine reasons for implant failure and to design novel techniques that improve primary stability and increase the quantity and quality of available bone [5,17]. Thanks to the development of new bone regeneration techniques and biomaterials, clinicians can now place implants in patients with bone deficits who would not previously have been candidates for this treatment [5,18]. Autogenous bone is the gold-standard bone graft due to its osteoconductive, osteogenic, and osteoinductive properties. However, it requires a second surgical field, with the associated morbidity, and a sufficient amount is not always available, leading to the utilization of other biomaterials as substitutes [19]. For instance, xenogeneic graft has proven to be a valid and safe alternative that obtains a similar bone gain to that achieved with autogenous bone [17]. Nonetheless, it is important to determine if this regenerated bone presents similar characteristics to native bone for implant rehabilitations. After the healing period, regenerated bone is expected to provide enough bone density and bone availability to obtain an adequate primary stability and its maintenance over time. A large number of studies have described ISQ values in non-regenerated bone, but there have been relatively few clinical studies on the primary and secondary stability of implants placed in bone regenerated with xenografts [12].

With this background, the objectives of this study were to compare primary and secondary stability values, using RFA, between dental implants placed in regenerated bone and those placed in non-regenerated bone, and to assess the influence of bone quality at the implant site on stability values. The null hypotheses were: (i) there is no difference in the stability of implants between those placed in regenerated bone and those placed in native bone of patients; and (ii) implant site bone quality affects primary and secondary stability in both native and regenerated bone.

## 2. Materials and Methods

### 2.1. Study Design

This study was designed as a prospective cohort study in accordance with the STROBE declaration for observational studies [20].

### 2.2. Patient Selection

The study population comprised consecutive patients receiving implant treatment for some degree of edentulism at the Masters of Oral Surgery and Implant Dentistry at the University of Granada between January 2018 and February 2019. Inclusion criteria were: age between 25 and 75 years; good oral and periodontal health; native bone with minimum healing period of 6 months post-exodontia or bone regenerated with xenograft 6 months earlier; sufficient bone availability for implants with length of 10 mm and diameter of 3.7 or 4.1 mm; and implants placed with a final insertion torque (IT) of 40 Ncm. Exclusion criteria were the presence of systemic disease, treatment with drugs that could compromise implant osseointegration, pregnancy, smoking habit of >10 cigarettes/day, poor oral hygiene, and the presence of periodontal disease (full-mouth plaque score and full-mouth bleeding score >20%).

### 2.3. Sample Size

The sample size was estimated for a confidence interval of 95% and statistical power of 90%, calculating a total sample size of 60 implants. Patients were assigned to two groups: 30 implants placed in native/non-regenerated bone ≥6 months post-exodontia (Group 1) and 30 implants placed in bone regenerated 6 months earlier with a xenograft (Group 2).

### 2.4. Pre-Surgery Phase

All patients gave their informed consent to participate after a detailed explanation of the aims of the study, the surgical procedure, and the follow-up period. Patients receiving the implants underwent a detailed clinical examination and exhaustive radiological study. Patients with implants in regenerated bone (Group 1) received a mineralized particulate bovine bone (Zimmer Biomet, Warsaw, IN, USA) and a resorbable collagen membrane (Zimmer Biomet, Warsaw, IN, USA) 6 months earlier in order to regenerate post-extraction sockets or any type of bone defects. The quantity and quality of bone at the implant site were evaluated using the classification of Lekholm and Zarb [21]. This classification is based on a radiographic assessment where higher density (D1–D2) is associated with a thick and homogeneous layer of cortical bone which surrounds a dense trabecular bone. Likewise, lower density (D3–D4) is associated with a thin layer of cortical bone surrounding a low-density trabecular bone [22]. The implant diameter was 3.7 or 4.1 mm in all patients according to their bone availability.

### 2.5. Surgical Phase

All implants were placed by the same surgeon (M.V.-R.), who always followed the same surgical procedure. Immediately before the surgery, patients performed a 2 min mouth wash with 10 mL 0.12% chlorhexidine and then received local anesthesia (4% articaine with 1:100.000 epinephrine). A full-thickness flap was raised by making a supracrestal incision extending towards adjacent teeth. The bone bed was then drilled in strict accordance with the instructions of the implant manufacturer about bone quality. After the drilling, Zimmer TSV implants (Zimmer Biomet, Warsaw, IN, USA) were placed at a final insertion torque of 40 Ncm, considered adequate to ensure primary stability and to prevent bone stresses [23]. The first stability measurement was then performed, followed by flap closure with Vicryl sutures (Ethicon, Inc., a Johnson & Johnson Company, Somerville, NJ, USA).

### 2.6. Stability Measurements

Implant stability was evaluated by RFA, using Osstell IDx (Osstell, Gothenburg, Sweden) to measure ISQ values. Two measurements (mesiodistal and bucco-lingual/palatal) were conducted per implant at baseline (implant placement), 8 weeks post-placement (second surgical phase), and 12 weeks (before prosthetic loading). All stability measurements were performed by the same operator (C.V.).

### 2.7. Statistical Analysis

IBM SPSS version 24.0 (SPSS Inc., Chicago, IL, USA) was used for the statistical analysis. In a descriptive analysis, means, standard deviations, standard errors, and median, minimum, and maximum values were calculated. The Shapiro–Wilk test was applied to check the normality of variable distribution and Levene’s test to assess the homoscedasticity. Student’s *t*-test was used to compare results for variables among the different factors, setting the level of significance at 0.05.

## 3. Results

### 3.1. Descriptive Analysis

Sixty implants were placed in 31 patients, 28 in males and 32 in females. The diameter was 3.7 mm for 24 implants and 4.1 mm for 36. The most frequent sites were posterior mandible (32 implants), and the least frequent sites were anterior maxilla (6 implants). The bone quality was D1–D2 for 36 implants and D3–D4 for 24 implants (Table 1).

### 3.2. Clinical Parameters

#### 3.2.1. Bone Type (Native vs. Regenerated)

**Figure 1:**
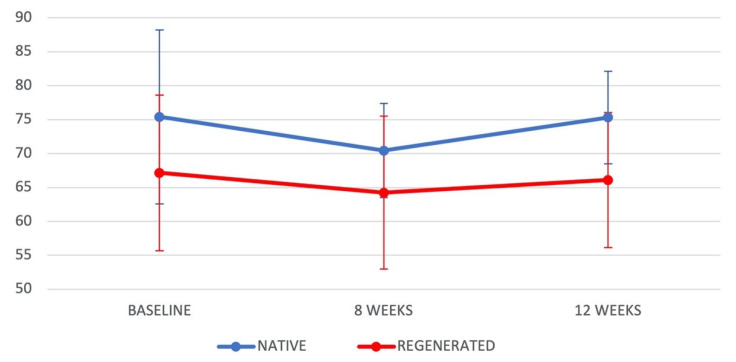
Evolution of ISQ values (mean ± standard errors) in Groups 1 and 2 at baseline, after 8 weeks, and after 12 weeks.

The ISQ was 75.40 ± 12.80 in native bone and 67.17 ± 11.47 in regenerated bone at baseline, 70.43 ± 6.93 in native bone and 64.23 ± 11.27 in regenerated bone at 8 weeks post-placement, and 75.33 ± 6.82 in native bone and 66.10 ± 8.72 in regenerated bone at 12 weeks (Table 2), when it reached similar values in both groups to those at baseline (Figure 1). The ISQ was significantly higher in native bone (always >70) than in regenerated bone (always >60) at all time-points, with *p*-values of 0.011 at baseline, 0.013 at 8 weeks, and <0.001 at 12 weeks (Table 2).

#### 3.2.2. Bone Quality (D1–D2 vs. D3–D4)

**Figure 2:**
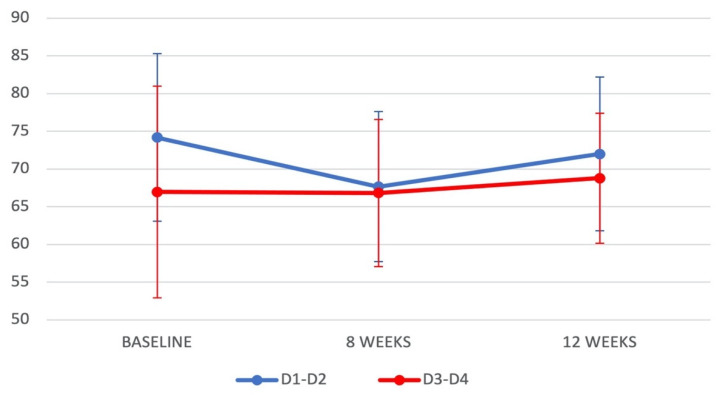
Evolution of ISQ values (mean ± standard errors) in bone D1–D2 and D3–D4 at baseline, after 8 weeks, and after 12 weeks.

At baseline, ISQ values were higher (*p =* 0.031) for implants placed in higher-density D1–D2 bone (74.17 ± 11.10) versus lower-density D3–D4 bone (66.96 ± 14.02). For implants placed in D1–D2 bone, ISQ values were lower at 8 weeks (66.83 ± 9.75) than at baseline but almost reached baseline levels at 12 weeks (72.00 ± 10.18). For those placed in D3–D4 bone, ISQ values remained stable at 8 weeks and reached a maximum at 12 weeks (68.79 ± 8.63) (Table 3) (Figure 2). Differences in ISQ values as a function of bone quality (D1–D2 vs. D3–D4) were not statistically significant at either 8 or 12 weeks after implant placement (Table 3).

## 4. Discussion

To the best of our knowledge, few published studies have compared implant stability between implants placed in native and regenerated bone. Greater primary and secondary implant stability were found in implants placed in native bone than those placed in regenerated bone. D1–D2 bone attained greater ISQ values, supporting the influence of implant site bone quality in primary implant stability.

Significantly higher ISQ values were observed for implants placed in native versus regenerated bone at baseline (*p* = 0.011), 8 weeks (*p* = 0.013), and 12 weeks (*p <* 0.001); therefore, the first null hypothesis of this study was not met. Nevertheless, both the primary stability (ISQ > 55) and secondary stability (ISQ > 65) of the implants in regenerated bone, measured at baseline and 12 weeks, respectively, were always above levels considered acceptable [16]. In both types of bone, the stability at 12 weeks was adequate for prosthetic loading [16]. Likewise, Zita et al. 2017 [24] obtained higher ISQ values at all measurement time-points (baseline and after 15, 30, 45, and 60 days) for implants placed in native versus regenerated bone, and also found that the values in regenerated bone were adequate for prosthetic loading. In contrast, in the study by Degidi et al. 2007 [25], although high RFA values were reported in both regenerated and non-regenerated bone, stability measures were higher in bone regenerated 6 months earlier with a xenogeneic graft at insertion time; however, there was a large difference in the sample size between those placed in regenerated (*n* = 63) and those placed in native bone (*n* = 17), which included four implants inserted immediately post-extraction. Moreover, Deli et al. 2014 [26] obtained higher ISQ values for implants placed in regenerated versus non-regenerated bone, but only when the regenerated bone had undergone a healing period of at least 12 months. When they compared implants inserted in native bone versus those placed in regenerated bone with a healing period of 6 months, ISQ values were lower in implants inserted in regenerated bone than for implants inserted in native bone, but were sufficient to start prosthetic loading, in line with the present findings of our study [26]. In general terms, with our results added to the literature reviewed, it can be concluded that xenogeneic grafts are valid alternatives in patients with bone deficit and a doubtful prognosis regardless of its comparison with native bone. Implants placed in the regenerated bone are sufficiently stable for successful prosthetic loading [19].

This result can only be expected when all implants in regenerated bone are inserted at six months after grafting. In our study, bone grafting was performed using mineralized particulate bone of bovine origin with a resorbable collagen membrane, known to promote bone regeneration and soft-tissue stabilization, and to preserve pre-extraction alveolar crest dimensions when grafted in post-extraction sockets [27,28,29,30,31]. No consensus has been established on the ideal healing time for regenerated bone before implant placement. A 6-month healing period was selected in order to reduce the waiting time before prosthetic loading and based on the results published by Degidi et al. 2007 [25] and Di Lallo et al. 2014 [32], who concluded that 6 months was sufficient to ensure adequate graft maturation and primary implant stability. Nevertheless, the selection of a single healing period was a study limitation, preventing comparisons with the stability of implants inserted after a longer time (e.g., 9 or 12 months post-grafting). All implants in native bone were inserted after a healing period of at least six months post-exodontia, previously described in the literature as an adequate time interval before implant placement [33].

It is known that implant stability is affected by many other parameters; however, the most relevant and determinant has been shown to be the bone quality [34]. In the present study, primary stability was significantly higher (*p* = 0.031) in D1–D2 versus D3–D4 bone. Nevertheless, the bone quality did not significantly influence the secondary stability at 8 or 12 weeks post-placement. In accordance with our findings, Sim et al. 2010 [35] reported that D3–D4 bone obtained lower ISQ values, but implant stability in this group increased significantly after 8–12 weeks, reaching ISQ values of over 70 and similar to the D1–D2 group. Moreover, O’Sullivan et al. 2004 [36], Yoon et al. 2011 [37], and Deli et al. 2014 [26], based on their investigations, all reported that bone quality was an important factor that influenced the primary stability of implants. In contrast, Farré-Pagès et al. 2011 [38] found no statistical significance between implant primary stability and bone quality, but it was analyzed in terms of insertion torque. When they measured primary stability using RFA, they found statistical significance and concluded that primary stability depends on bone quality and implant location. Different conclusions were obtained by Herekar et al. 2014 [39], where they found that bone quality was correlated with secondary stability but not with primary stability. These findings could be due to the fact that secondary stability was measured 4 weeks post-placement. Huang et al. 2020 [11] reported that the reason for these controversial views in the literature may be the subjective and inaccurate method used to determine bone quality; thus, the classification of bone quality in investigations remains variable [40].

### Strengths and Limitations of the Study

RFA is widely used in the assessment of implant stability. Most of the research reviewed demonstrates the influence of other factors in primary stability, such as IT [5,41,42,43]. One advantage of this study was the standardization of the surgical protocol, in order to avoid the effect of different IT in our results. The IT was always 40 Ncm, following numerous reports in the literature recommending this level of torque to achieve adequate primary stability [11,44,45,46,47]. Likewise, the implant length was always 10 mm, and it is well documented that implants ≥10 mm provide good stability outcomes [48,49,50,51,52] and allow immediate loading with satisfactory results [53,54,55]. The same brand and type of implant was placed in all patients in order to avoid differences attributable to variations in thread design, body shape, or implant surface [3,56,57]. The main limitation of this study was the relatively small sample size, although it proved adequate for yielding statistically significant results. There was only one healing period in regenerated bones; however, 6 months has been proven to be enough time to obtain adequate primary stability [33]. Finally, the authors encourage future researchers to perform more clinical trials on the main objectives of this study to result in more conclusive statements.

## 5. Conclusions

Based on these findings, it can be affirmed that higher ISQ values are obtained in implants placed in native bone than in bone regenerated with xenograft but are always >60 in the latter, considered to indicate adequate implant stability. In both types of bone, the primary stability of implants was superior in denser bone (D1–D2 versus D3–D4), but the secondary stability was not significantly affected by bone quality at the implant site. 

## Figures and Tables

**Table 1:** Data on patient sex, implant diameter, implant distribution, and bone quality.

		Implants	
		(*n*)	(%)
Sex	Male	28	46.7
	Female	32	53.3
Implant diameter	3.7	24	40
	4.1	36	60
Implant site	Maxillary anterior	6	10
	Maxillary posterior	17	28.3
	Mandibular anterior	5	8.3
	Mandibular posterior	32	53.3
Bone quality *	D1–D2	36	60
	D3–D4	24	40

* Lekholm and Zarb classification.

**Table 2:** Between-group comparison of ISQ values at baseline, 8 weeks, and 12 weeks.

Bone Type							
		*n* (Mean ± SD)	Standard Error	Median	Minimum	Maximum	*p*-Value
ISQ Baseline	Native	30 (75.40 ± 12.80)	2.34	76.50	25.00	91.00	0.011 *
	Regenerated	30 (67.17 ± 11.47)	2.09	64.50	47.00	85.00	
ISQ 8 weeks	Native	30 (70.43 ± 6.93)	1.26	71.00	57.00	83.00	0.013 *
	Regenerated	30 (64.23 ± 11.27)	2.06	62.50	44.00	88.00	
ISQ 12 weeks	Native	30 (75.33 ± 6.82)	1.25	75.50	63.00	87.00	0.000 *
	Regenerated	30 (66.10 ± 9.93)	1.81	67.00	49.00	85.00	

SD = standard deviation. * Student’s *t*-test.

**Table 3:** ISQ values as a function of bone quality at baseline, 8 weeks, and 12 weeks.

Bone Quality							
		*n* (Mean ± SD)	Standard Error	Median	Minimum	Maximum	*p*-Value
ISQ Baseline	D1–D2	36 (74.17 ± 11.10)	1.85	73.50	49.00	91.00	0.031 *
	D3–D4	24 (66.96 ± 14.02)	2.86	70.00	25.00	84.00	
ISQ 8 weeks	D1–D2	36 (67.67 ± 9.93)	1.66	70.00	44.00	87.00	0.750 *
	D3–D4	24 (66.83 ± 9.75)	1.99	67.50	50.00	88.00	
ISQ 12 weeks	D1–D2	36 (72.00 ± 10.18)	1.70	73.50	50.00	87.00	0.209 *
	D3–D4	24 (68.79 ± 8.63)	1.76	70.50	49.00	81.00	

SD = standard deviation. * Student’s *t*-test.

## Data Availability

The data presented in this study are available on request from the corresponding author.
